# Supplementary material for: Targeted Recovery as an Effective Strategy against Epidemic Spreading
Source: Sci Rep. 2017 Oct 30;7:14356. doi: 10.1038/s41598-017-14763-5 (PMC5662666; doi:10.1038/s41598-017-14763-5)
Supplement: Supplementary file 1 — Supplemental Material [file 41598_2017_14763_MOESM1_ESM.pdf]

Supplemental Material: Targeted Recovery as an Effective Strategy against Epidemic Spreading

L. Böttcher,[1,*] J. S. Andrade Jr.,[2] and H. J. Herrmann[1,2]

[1]*ETH Zurich, Wolfgang-Pauli-Strasse 27, CH-8093 Zurich, Switzerland*

[2]*Departamento de Física, Universidade Federal do Ceará, 60451-970 Fortaleza, Ceará, Brazil*

(Dated: October 9, 2017)

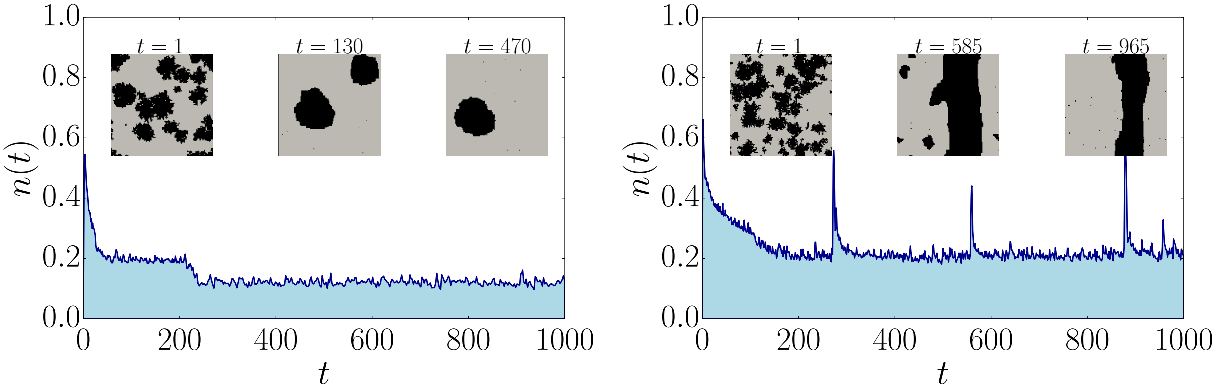

Figure S1. **Random initial conditions.** A small spontaneous infection term $p > 0$ leads to multiple and randomly distributed spreading seeds indicated by the black lattice sites in the insets. (left) The time evolution of $n(t)$ for $r = 10$ and $p = 0.01$. (right) The time evolution of $n(t)$ for $r = 10$ and $p = 0.1$. Both simulations have been performed on a square lattice with $N = 128 \times 128$ nodes. Due to spontaneous infections, small confined epidemic spots form and disappear after some time resulting in peaks in the time evolution of $n(t)$, cf. inset for $t = 585$.

RANDOM INITIAL CONDITIONS

In Fig. 1 of the main text we show that our targeted recovery protocol leads to a confined epidemic spot on a spatially embedded network assuming a single circular spreading seed. Here we demonstrate that this effect is still observable in the case of multiple and randomly distributed spreading seeds. We therefore perturb our dynamics with spontaneous infections ($p > 0$). In Fig. S1 we illustrate the effect of spontaneous infections. Initially, many spreading seeds form and some vanish spontaneously whereas others merge at some point. Up to fluctuations due to the spontaneous infections, we find that the epidemic approaches a stationary state in which it stays confined.

DYNAMICS ON A RANDOM GEOMETRIC GRAPH

This section is dedicated to the analysis of our model's properties on a random geometric graph [1]. In Fig. S2 (left), we show the epidemic transitions for different values of ϵ, i.e. different proportions of random and targeted interventions. We find that our targeted recovery strategy with $\epsilon = 1$ also outperforms random interventions where $\epsilon < 1$ on a random geometric graph. These results are in accordance with the transition characteristics

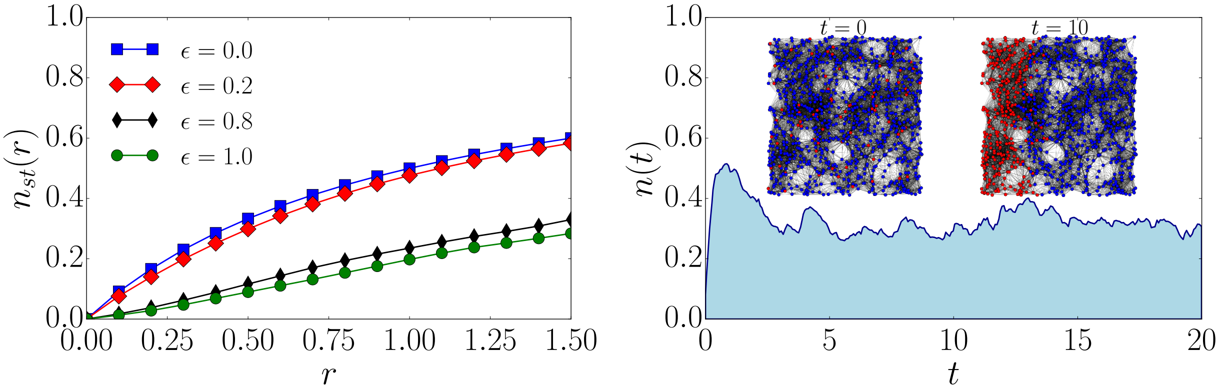

Figure S2. **Dynamics on a random geometric graph.** (left) The fraction of infected nodes in the stationary state $n_{st}(r)$ as a function of r for different values of ϵ. All simulations have been performed on a random geometric graph with $N = 10^4$ nodes, a radius of 0.125 and $n(0) = 0.13$ [1]. The targeted recovery strategy with $\epsilon = 1$ outperforms random interventions where $\epsilon < 1$. The data are averaged over 250 samples. (right) The time evolution of $n(t)$ for $r = 2$, $\epsilon = 1$ and $n(0) = 0.08$ on a random geometric graph with $N = 10^3$ nodes and a radius of 0.125. Red nodes correspond to infected ones.

that have been found for embedded networks without and with long-range connections, cf. Figs. 5 and 6 in the main text.

Based on Fig. S2 (right), we also conclude that the epidemic stays spatially confined on a random geometric graph as in the case of the spatially embedded network studied in the main text, cf. Figs. 2 and 5 in the main text. The insets of Fig. S2 (right) show the initially unconfined epidemic which later on gets confined in the left part of the network.

TRANSITION DUE TO SPONTANEOUS RECOVERY

In the main text, we have shown that confined epidemics still emerge for substantial amounts of random recovery events. However, if these random perturbations are too large, the confinement is no longer observable. In Fig. 5 of the main text, we illustrate the stationary density of infected nodes $n_{st}(r)$ as well as the number of infected nodes as a function of r for different values of ϵ. Two different representations have been used since $n_{st}(r)$ is the appropriate order parameter for small values of ϵ where the number of infected nodes is proportional to the system size. However, for values of ϵ close to one, the epidemic sport

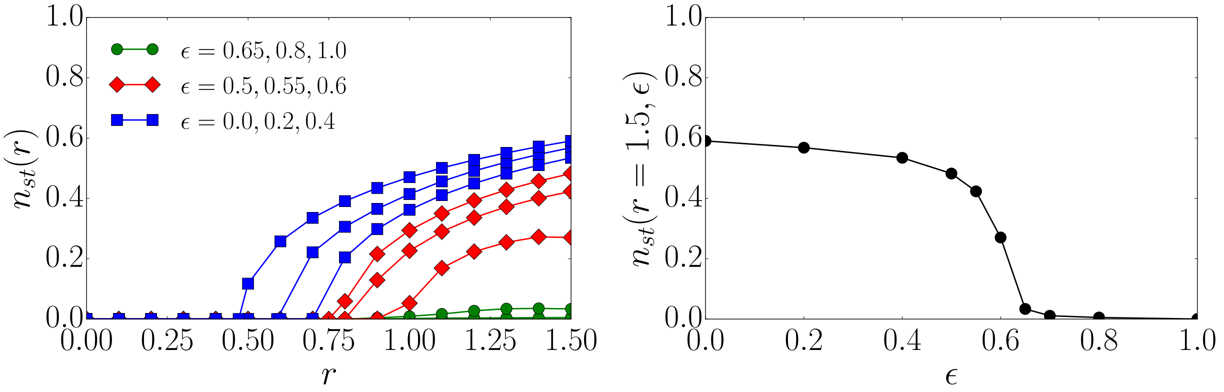

Figure S3. **Transition due to spontaneous recovery.** (left) The dependence of $n_{st}(r)$ on r for different values of ϵ. The blue squares represent the case where $\epsilon \in \{0.0, 0.2, 0.4\}$, the red diamonds $\epsilon \in \{0.5, 0.55, 0.6\}$ and the green circles $\epsilon \in \{0.65, 0.8, 1.0\}$. The data for $\epsilon \in \{0.65, 0.8, 1.0\}$ are averaged over 500 samples. The threshold values separating the absorbing and the active phase increase with ϵ. All simulations have been performed on a square lattice with $N = 1024 \times 1024$ nodes. (right) The stationary density $n_{st}(r = 1.5, \epsilon)$ for $r = 1.5$ as a function of ϵ. Small values of n_{st} are found for ϵ larger than 0.6 what suggests the onset of the confinement effect.

has a characteristic length scale as shown in Fig. 1 of the main text and the number of infected nodes does not depend on the system size. To allow for a better comparison of the transition curves for different values of ϵ, we only focus on the densities $n_{st}(r)$ as a function of r in Fig. S3 (left). We clearly see that the threshold values separating the absorbing disease free and the active phase increase with ϵ. Furthermore, for values of ϵ larger than 0.6 the dynamics enters the confinement regime where the densities approach small values, cf. Fig. S3 (right).

INFLUENCE OF LARGE SPREADING RATES AND RANDOM MIXING

In the main text, we described the transition from a lattice to a random network by introducing a characteristic link length ζ on the graph [2]. In Fig. S4 (left), we illustrate the difference $\Delta n_{st}(r) = n_{st}(r, \epsilon = 0) - n_{st}(r, \epsilon = 1)$ as a function of r for different values of ζ. We clearly see that $\Delta n_{st}(r)$ tends to zero for large values of r implying that targeted recovery ($\epsilon = 1$) and random intervention ($\epsilon = 0$) lead to same densities of infected nodes on such networks as r increases. The intuition behind this effect is the reduced number of neighborhoods with

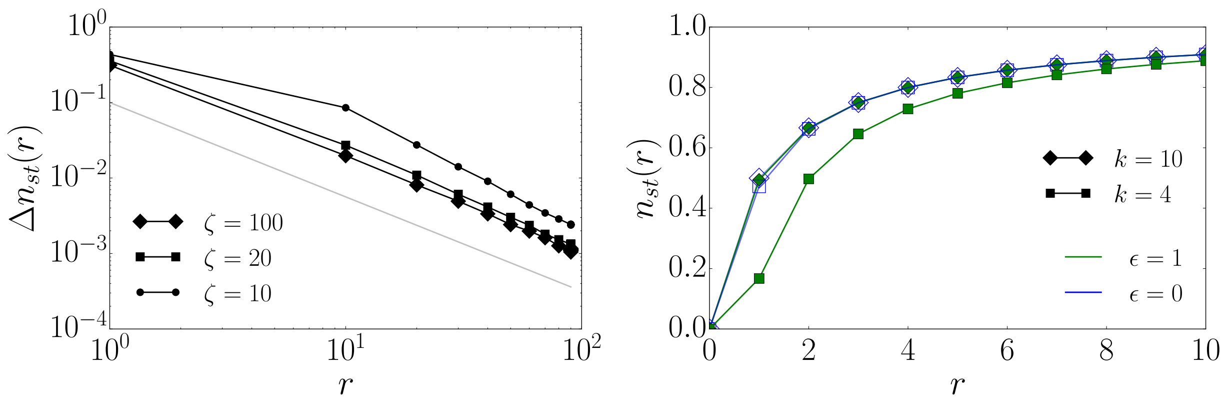

Figure S4. **Influence of large spreading rates and random mixing.** (left) The difference $\Delta n_{st}(r) = n_{st}(r, \epsilon = 0) - n_{st}(r, \epsilon = 1)$ as a function of r on a network with $N = 128 \times 128$ nodes and degree $k = 4$ for different characteristic link lengths ζ. The targeted recovery scheme converges towards the non-targeted case for large values of r. The grey solid line is a guide to the eye with slope -1.25. (right) The fraction of infected nodes in the stationary state $n_{st}(r)$ as a function of r on two random regular graphs with $N = 10^5$ nodes and degrees $k = 4$ and $k = 10$ respectively. The targeted strategy with $\epsilon = 1$ (green filled markers) tends towards the random recovery protocol where $\epsilon = 0$ (blue hollow markers) for large values of r and k.

only a few infected neighbours in the case of large values of r. Similarly, one would expect that targeted and random recovery are indistinguishable for random regular graphs with large degree since random mixing hinders targeted intervention. To study this effect, we show the density of infected nodes $n_{st}(r)$ as a function of r for two random regular graphs with $N = 10^5$ nodes and degrees $k = 4$ and $k = 10$ in Fig. S4 (right). We find that targeted and random intervention lead to almost the same densities for the random network with $k = 10$. In the case of $k = 4$, the two protocols coincide for large spreading rates r.

* lucasb@ethz.ch

[1] M. Penrose, *Random geometric graphs* (Oxford University Press, 2003).

[2] M. M. Danziger, L. M. Shekhtman, Y. Berezin, and S. Havlin, EPL **115**, 36002 (2016).
